# Why Most Australians Consider It Valuable to Find Harmless Abnormalities with Diagnostic Tests: A Mixed-Methods Study

**DOI:** 10.1177/0272989X251413288

**Published:** 2026-01-30

**Authors:** Tomas Rozbroj, Ming Hui Hoo, Alexandra Gorelik, Denise A. O’Connor, Rachelle Buchbinder

**Affiliations:** School of Public Health and Preventive Medicine, Monash University, Melbourne, VIC, Australia; School of Public Health and Preventive Medicine, Monash University, Melbourne, VIC, Australia; School of Public Health and Preventive Medicine, Monash University, Melbourne, VIC, Australia; School of Public Health and Preventive Medicine, Monash University, Melbourne, VIC, Australia; School of Public Health and Preventive Medicine, Monash University, Melbourne, VIC, Australia

**Keywords:** attitude, diagnosis, incidental findings, overdiagnosis, public, risk assessment

## Abstract

**Background:**

Individuals commonly want diagnostic testing even after being informed the test is clinically unbeneficial and has risks. These preferences are poorly understood but may relate to beliefs that any testing information is valuable. To explore this, we examined Australian adults’ attitudes toward finding harmless abnormalities using diagnostic tests and the broader beliefs related to these attitudes.

**Methods:**

Data collected via survey were analyzed using mixed methods. Free text explaining attitudes to finding harmless abnormalities were analyzed using comparative content and interpretative analyses. Associations between attitudes to finding harmless abnormalities and broader beliefs and demographics were analyzed using regression.

**Results:**

Almost three-fifths of 655 participants considered it valuable to identify harmless abnormalities using tests. Qualitative analyses showed this attitude was driven by beliefs that identification would provide psychological reassurance, valuable biodata, and enable monitoring and management of the harmless abnormalities. These beliefs were underpinned by a skepticism that abnormalities can ever be harmless and by a range of beliefs about the broader value of diagnostic testing. Participants with negative attitudes to identifying harmless abnormalities were concerned about resultant anxiety and unnecessary health interventions. Regression showed that positive attitudes to identifying harmless abnormalities were associated with greater confidence in doctors, lesser concerns about overtreatment, and a stronger desire to know as much about their bodies as possible as well as with several demographic variables.

**Conclusions and Implications:**

Our study explores why people seek diagnostic tests that they know lack obvious clinical benefits. It identifies broader beliefs and psychological factors that profoundly influence testing choices. This knowledge will help overcome the limitations of existing strategies to explain the risks of tests to patients and the public.

**Highlights:**

- Findings help explain why facts showing that particular diagnostic tests are ineffective or harmful fail to dissuade many Australians from seeking those tests.
- Many Australians value diagnostic testing for perceived reassurance, understanding one’s body, and use in medical decision making.
- Many are skeptical that identifying incidentalomas is harmful, and are confident they can avoid unnecessarily treating them.
- Messages about testing risks should focus on broader beliefs and respond to psychological factors that undermine the effect of risk/benefit information.

Australians rely on diagnostic testing and medical screening. In 2022–2023, two-thirds of the population accessed at least 1 of the 196.9 million Medicare-subsidized diagnostic tests performed annually.^1^ Between two-fifths and three-quarters of Australians invited to 1 of the national preventative cancer screening programs participated.^2^ However, this confidence in testing can also motivate Australians to seek unnecessary tests^3^ and minimize or reject testing risks.^4^ Excessive testing is a significant problem: it is estimated that 30% of health care is useless and 10% harmful.^5^ Importantly, many individuals continue to want tests despite accessing risk/benefit information showing that having the test would be pointless or harmful.^3,6,7^

The ineffectiveness of risk/benefit information to persuade individuals to consider the risks and necessity of testing undermines efforts to reduce overtesting, affects shared decision making, and may lead to some patients having unnecessary and sometimes life-changing medical interventions.^8^ Public support is needed to help address this problem,^9^ but it is difficult to gain.^10^ For example, evidence-based changes to reduce excessive cancer screening have resulted in alienation and hostility among the public.^6,7^ The inefficacy of risk/benefit information also raises questions about clinical communication and patient decision aids. Decision aids are intended to support patient decisions, but they overwhelmingly rely on explaining risks/benefits to do this.^11^ Doctors report difficulties in dissuading some patients from seeking unnecessary tests and may inappropriately request tests to maintain trust and provide reassurance^12^ (with limited effect^13^). For example, in reducing unnecessary imaging for low-back pain—one of the most overused tests globally^14^ and in Australia^15^—patient demand for imaging has been a challenge for doctors and in the design of interventions.^16^

The effectiveness of risk/benefit information appears to be undermined by individuals’ broader beliefs about testing and disease, psychosocial factors, emotions, and biases. A reanalysis of qualitative studies suggested that individuals problematize the concept of “inappropriate tests,” because they believe tests provide reassurance and useful biodata.^3^ Fear of disease, uncertainty, and anticipated regret about “not acting” may drive tendencies to seek tests even if they are risky.^17,18^ A range of biases and schemas prime individuals to test; for example, tests provide individuals with an illusion of control.^19^ Conversely, ingrained confidence in testing can trigger cognitive dissonance toward messages dissuading testing.^20^ Attempts to deimplement unwarranted testing can make people feel devalued, discriminated against,^6,7^ suspicious about ulterior motives such as cost cutting,^6^ and provoke anxiety.^17^

While research has explored perceptions of diagnostic testing risks and benefits, there is limited empirical work examining how individuals perceive testing beyond its role in detecting specific disease. Existing insights primarily come from qualitative studies,^3^ leaving a need for quantitative and mixed-methods research. Most insights come from studies that focused on the risk and benefit of detecting specific pathology, for example, women’s perceptions of risks and benefits in breast screening.^21^ The limited evidence suggests that these reasons for testing beyond finding disease do inform testing choices.^3,7,22^ They also inform responses to communications about these choices. For example, individuals may value incidental findings and reject that these lead to overtreatment,^3^ which potentially undermines messages cautioning against unnecessary testing to avoid incidental findings.^23^ To gain a more comprehensive understanding and overcome some of the existing limitations, a study explicitly focused on the value of diagnostic tests beyond identifying disease is needed.

The aim of this study was to assess Australian adults’ attitudes about identifying harmless abnormalities using diagnostic tests and examine their broader beliefs related to these attitudes.

We hypothesized that exploring attitudes toward finding harmless abnormalities on tests would uncover belief systems associated with preferences for unnecessary testing. We expected that this knowledge can be harnessed to address the factors that undermine risk/benefit information in informing testing choices, including in decision aids, public/patient messaging, and clinical communications.

## Methods

This study was reported according to Good Reporting of A Mixed Methods Study (GRAMMS) checklist,^24^ included in Appendix 1.

### Study Design

Cross-sectional online survey of Australian adults.

### Survey Instrument

Measures comprised 15 items, detailed in Appendix 2 and summarized below. The measures examined attitudes to identifying harmless abnormalities on tests (2 items), broader attitudes/beliefs (4 items), and demographics/self-rated health (9 items).

The attitude to identifying harmless abnormalities were examined using 2 questions following this prompt:

In some cases, tests find abnormalities in the body that would not go on to cause any harm. For example, they find lumps, elevated readings or physical changes that would not cause symptoms or complications. If they weren’t detected, the patient would never find out that they had them.

We then asked, “How valuable or harmful do you think it is to identify abnormalities that would never harm patients?” First, we sought responses on a 5-point Likert scale (*very valuable* to *very harmful*). Second, we sought free-text explanations of the Likert responses. Gist theory posits that individuals conceptualize the general meaning of information rather than detailed, precise data.^25^ With regard to diagnostic testing, individuals tend to conceptualize whether their results are “normal” or “not” and whether the test was worthwhile.^10,26^ The scale wording approximated this simplified, bottom-line understanding of testing. The question wording “abnormalities that would never harm patients” was chosen to communicate a result that is “not normal but harmless.”

Broader attitudes/beliefs were measured by four 5-point Likert questions, examining the following:

- attitude regarding whether the more one knows about their body the better,
- beliefs about the extent that harmless abnormalities found on tests are unnecessarily treated,
- beliefs about the extent that doctors can distinguish harmful abnormalities found on tests from harmless ones, and
- beliefs about the extent that patients can avoid unnecessary treatments for harmless abnormalities found on tests, if they take appropriate steps.

The 9 demographic variables included age; Australian state/territory of residence; whether participants lived in a major city, regional, or rural area; gender; whether participants had children; highest educational attainment; household income; whether participants are or were health care workers; and self-rated health (General Self-Rated Health scale, comprising 5 points between 1 = *poor* and 5 = *excellent*, where higher scores indicate greater perceived healthiness^27^).

The present article reports on a subset of data from a larger survey that examined Australians’ beliefs about the risks of diagnostic testing. As part of this survey, prior to any of the questions used in the present study being asked, respondents were invited to view a video suggesting that it is important to ask doctors about the necessity, efficacy, and safety of medical interventions.^28^

The survey instrument was pilot tested and refined among the research team, which included clinician researchers, qualitative experts, an implementation scientist, and a statistician.

### Participants, Sample Size, and Recruitment

We sampled adults (aged 18+ y) living in any state/territory of Australia. At least 400 complete responses were calculated to be required for the quantitative analysis with a ±5% margin of error. The sample target was set at *N*≥650 to account for incomplete survey responses, which problematize multivariate regression analysis.

We recruited between January and April 2022. We targeted Meta (Facebook) users via paid advertisements^29^ delivered in quotas to groups stratified by age (>50/≤50 y) and gender (men/women) to achieve a more balanced sample. This stratification reflected that many diagnostic tests are offered from age 50 y in Australia, and different tests are offered to men and women.^30^ All advertisements directed users to the Qualtrics survey landing page via a generic link.

### Analysis

Analyses comprised qualitative content and interpretative analyses of free-text responses compared according to attitudes to identifying harmless abnormalities and multivariate regression analysis of associations between attitudes to identifying harmless abnormalities and other fixed-response variables.

#### Methodology

A mixed-methods approach was selected to gain a more comprehensive understanding^31^ of different attitudes/beliefs related to identifying harmless abnormalities on diagnostic tests. Text data favored discovering unanticipated findings. Analyzing it using content analysis paired with interpretative analysis prioritized a comparative approach and explanatory insights, respectively.^32^ The regression allowed us to systematically study associations between attitudes to identifying harmless abnormalities and broader factors believed to relate to seeking unnecessary testing: maximalist tendencies,^33^ beliefs about the inherent value of information derived from testing,^34^ an underestimation of testing harms,^35^ and an overconfidence in personal ability to negotiate testing choices.^3^

We approached the text analysis from an “emergent coding” perspective.^36^ This fit our relatively descriptive methods, lack of literature to inform our analytic frame, and the aim of developing a narrative addressing the research aim.^37^

To suit our survey modality, a convergent data integration design was selected, meaning that qualitative and quantitative data were collected concurrently.^38^ Integration occurred at 1) the analysis stage, in linking text responses to descriptive statistics to inform comparison of qualitative results, and 2) at the interpretation stage, the discussion where the combined findings were considered.

#### Content and interpretative analyses

Content analysis examined beliefs explicitly expressed by participants (e.g., comments stating they wanted to identify and monitor harmless abnormalities in case these require treatment in the future). We de-coupled comments from other data. One author (M.H.H.) iteratively read these and developed a code frame, then coded the whole dataset. A sample of >10% of coding was checked by a second author (T.R.) during this process, and disagreements were discussed. Comments could be coded to more than 1 code. Homogenous groups of content were organized into themes by M.H.H. and T.R.^32^ Themes were checked for internal homogeneity.

Interpretative analysis examined potential underlying beliefs that may explain explicit beliefs captured by content analysis (e.g., the example above seemed to imply a belief that even “harmless” abnormalities can cause harm). We adapted our approach from “latent” coding in content analysis^32^ to focus on underlying beliefs. T.R. examined data in each content analysis theme for underlying beliefs, iteratively developing a code frame. He then coded all data captured by content analysis themes and generated interpretative themes.

Likert answers to “how valuable or harmful do you think it is to identify abnormalities that would never harm patients?” were cross-tabulated with the themes from both phases of qualitative analysis to identify differences in explicit themes and underlying beliefs related to attitudes toward identifying harmless abnormalities.

We selected exemplary comments that typified theme/s, but that were longer than average, to exemplify several themes at once.

#### Regression

The regression model examined the predictors of attitudes toward identifying harmless abnormalities using tests. To ensure a consistent sample across mixed methods, only data from participants who provided free-text responses were included in quantitative analysis.

We checked data to ensure no individual completed the survey multiple times. We considered comparing complete/incomplete responses. However, a large majority of those who did not complete the survey answered only the first few questions; therefore, comparison would be meaningless. We generated descriptive statistics as *n* (%) for categorical data and mean (SD) for continuous data.

We examined univariate associations between the Likert response about attitudes to identifying harmless abnormalities (primary outcome) and other fixed-response variables. Either χ^2^, Fisher’s exact test, or *t* test were used, as appropriate.

Factors associated with attitudes to identifying harmless abnormalities at the *P* < 0.15 level were entered into multivariate ordered logistic regression models (ordered: valuable, ambivalent, harmful), with beliefs as predictors and demographics as controls. Only participants who answered on all variables in the model were included. All assumptions for ordered logistic regression as well as potential multicollinearity between selected predictors were tested prior to the analysis. Due to the nature of the data (self-report), missing data were not imputed, and the analysis was conducted using a complete data approach. Different combinations of control variables that were significant at *P* < 0.15 in the univariate analysis were tested for model fit, examined using Akaike (AIC) and Bayesian (BIC) information criteria.^39^ Associations at *P* < 0.05 were considered significant.

### Ethics

This study was approved by the Cabrini Research Governance Office (07-04-11-21) and Monash University Human Research Ethics Committee (31176).

## Results

For brevity, we refer to participant groups as “all” (undifferentiated by attitude to identifying harmless abnormalities) or “valuable”/”harmful”/”ambivalent” (indicating specific attitude to identifying harmless abnormalities).

Comments were entered by 723 participants. However, 3 comments could not be analyzed (e.g. “no”), and a further 65 participants appeared to misunderstand the instruction to rate their attitudes toward identifying harmless abnormalities. We excluded these 68 participants, resulting in a sample of *N* = 655. The aggregate text comments comprised 17,796 words (mean word count = 27, SD = 26).

### Participant Characteristics and Attitudes/Beliefs

Participant characteristics and attitudes/beliefs are reported in Table 1. Most participants lived in metropolitan areas. Men and women responded in similar numbers (mean ± SD, age = 55 ± 19 y). Two-thirds had a bachelor’s degree or higher, and a fifth were health workers at some stage (currently, retired, or changed profession).

**Table 1:** Participant Characteristics and Attitudes/Beliefs (*N* = 655)

Demographics	*n*	%^a^
State or territory of residence		
Australian Capital Territory	18	3
New South Wales	147	22
Northern Territory	6	1
Queensland	111	17
South Australia	37	6
Tasmania	23	4
Victoria	242	37
Western Australia	71	11
Gender		
Female	314	48
Male	317	48
Other	19	3
No answer	5	1
Age, y		
18–35	142	22
36–55	142	22
56–75	296	45
76 or older	75	11
Have children		
No	276	42
Yes	377	58
No answer	2	0
Remoteness of residence		
State/territory capital city	420	64
Regional area	219	33
Remote area	16	2
Health care worker		
No	505	77
Yes	131	20
No answer/unclear^b^	19	3
Highest level of educational attainment		
Higher learning certificate/diploma or less	214	33
Undergraduate degree	214	33
Postgraduate degree	226	35
No answer	1	0
Weekly household income, A$		
Low (<1,000)	174	27
Mid (1,000–<3,000)	190	29
Mid-high (3,000–<4,000)	35	5
High (4,000+)	36	5
No answer	220	34
Self-rated health		
Excellent	49	7
Very good	222	34
Good	232	35
Fair	115	18
Poor	37	6
Attitude to identifying harmless abnormalities on tests	*n*	%^a^
Very valuable	138	21
Somewhat valuable	228	35
Ambivalent	160	24
Somewhat harmful	114	17
Very harmful	15	2
Broader attitudes/beliefs	*n*	%^a^
The more I know about my body the better		
Strongly agree	435	66
Somewhat agree	177	27
Disagree or neutral	41	6
No answer	2	0
How commonly are harmless abnormalities unnecessarily treated?		
Commonly	386	59
Uncommonly	180	27
Don’t know	88	13
No answer	1	0
Extent that health care professionals can distinguish between harmless/harmful abnormalities detected on tests?		
Usually yes	481	73
Usually not	127	19
Don't know	47	7
Extent that patients who take the “right steps” can avoid unnecessary treatment on abnormalities detected on tests?		
Usually yes	491	75
Usually not	129	20
Don’t know	35	5

a Due to rounding, not all percentages sum to 100.

b Participants could choose from several health care worker categories (e.g., “nurse”) or “other” and enter free text. Free text was back coded, as some participants described professions that we did not consider to be health work (e.g., security at hospital). “Unclear” refers to a few instances in which we could not determine based on free text whether participants who answered “other” were health workers.

Participants usually considered it valuable to find harmless abnormalities on tests. Almost all agreed it is good to know as much about their body as possible. While three-fifths believed that harmless abnormalities commonly get overtreated, three-quarters had confidence in both doctors’ abilities to distinguish such abnormalities and patients’ abilities to avoid overtreatment.

### Content Analysis

There was 92% coding consensus between the primary coder (M.H.H.) and code checker (T.R.).

We identified 4 themes, which captured comments from 594 participants. Themes are summarized in Table 2. This table also compares by group attitude the percentage of comments coded to each theme (e.g., 26% of the 334 comments from the “valuable” group were coded to theme C1). Table 3 provides exemplary comments paired with key demographic information.

**Table 2:** Coding Comparison by Group and Content Analysis Theme

Content Analysis Theme	% of Comments per Attitude Group Coded to Each Theme (*n* = 594)^a^		
	Harmful (*n* = 124)^b^	Ambivalent (*n* = 136)^b^	Valuable (*n* = 334)^b^
1. Psychological impacts of identifying harmless abnormalities (*n* = 231)	76%	37%	26%
a. Psychological harms of identification (*n* = 117)	71%	13%	4%
b. Psychological benefits of identification (*n* = 58)	0%	2%	16%
c. Depends (*n* = 60)	6%	21%	7%
2. Reasons to identify harmless abnormalities (*n* = 345)	8%	37%	85%
a. Good to be informed (*n* = 177)	4%	23%	42%
b. Knowledge will assist management of abnormality (*n* = 177)	4%	13%	46%
3. Reasons to avoid identifying harmless abnormalities (*n* = 93)	52%	15%	2%
4. Context is key for judging value of identifying harmless abnormalities (*n* = 87)	8%	46%	4%

a Comments were multicoded and not all were attributable to subthemes. Not all comments contributed to final themes: subtheme *n*≠ theme *n*; sum of theme *n*≠*N*; sum of comments coded to themes ≠*N*.

b *n* of comments per group.

**Table 3:** Exemplary Comments

Attitude Group	Comment	Content Analysis Theme	Interpretative Analysis Theme
Valuable	“The known abnormalities will not be cause of worry nor further investigation if there is emergency imaging.” —69-y-old male with undergraduate degree, no professional health care experience, good health	1	1
Harmful	“In my experience it has led to more tests, with added cost, potential harm and stress, to prove nothing. Jumping at shadows is how my late husband described it during his cancer journey. There were many!” —72-y-old female with postgraduate degree, allied health work experience, very good health	1, 3	3
Harmful	“If you find things out, even if they aren’t harmful, you can monitor for changes in the future, in case it does become harmful. But you can also be reassured, if you’d found it out yourself there might have been more worry, but you can also be less worried if you find similar, non-harmful things in the future.” —36-y-old female with undergraduate degree, no professional health care experience, good health	1, 2	1, 2
Harmful	“Being told of any abnormality can cause anxiety. It would raise concerns about how certain it is that the abnormality will never be harmful. It depends on what is found. Most people would be OK if the abnormality was commonly known as not being harmful. However, lesser known or ‘scary’ abnormalities (e.g., lumps, heart irregularities) are a different story.” —66-y-old male with undergraduate degree, no professional health care experience, good health	1, 4	2, 3
Valuable	“If they won’t go on to harm you then they are just part of the general information about your body and set some benchmarks. However, it could be that information and attitudes change in the future and the information could become useful and valuable.” —77-y-old female with postgraduate degree, nursing/midwifery experience, very good health	2	2
Valuable	“It leaves the individual with the information about their body and then they can decide whether or not to follow it up with treatment regardless if it is necessary. It also gives the doctors/nurses more exposure to the lumps to be able to identify them in other patients more easily and to be able to tell them if it’s serious enough to be treated.” —23-y-old male with secondary school education, no professional health care experience, very good health	2	1, 2
Harmful	“Could cause someone to worry unnecessarily. It may also be at significant cost in terms of both money and time. It may also just mean the doctor/medical practice makes money for no benefit to the patient.” —60-y-old female with postgraduate degree, no professional health care experience, very good health	1, 3	3
Ambivalent	“It honestly depends on context. On one hand it could cause the patient anxiety and/or possibly result in unnecessary treatment that ends up being more harmful than just leaving it. On the other, not having tests because of the chance of finding something benign, could mean something harmful might be missed. Not to mention the chance of said benign finding possibly becoming harmful in the future and can now be monitored. Additionally, the previous page of questions are way too vague in terms of what diagnostic tests/screenings they’re referring to. There’s a big difference in the risk/benefit ratio between tests that don't use ionising radiation (e.g., ultrasound) and tests like mammograms and CTs [computed tomography scans] which do, and can’t be grouped into one vague question.” —25-y-old nonbinary with postgraduate degree, no professional health care experience, fair health	1, 2, 3, 4	1, 3
Ambivalent	“For some people there would be significant harm, due to health anxiety or an insistence on overtreatment. For others there is significant value in being able to say ‘oh, I know that’s there—no need to worry about it’ and in satisfying their own curiosity. Unfortunately, incidental findings just happen, and we can’t predict which group of people they happen to.” —43-y-old female with postgraduate degree, no professional healthcare experience, good health	1, 4	2, 3

#### C1. Psychological effects of identifying harmless abnormalities

Participants from all groups focused on the impact of identifying harmless abnormalities on their psychological well-being. However, the perceptions of that impact varied between groups. Comments from the valuable group tended to describe psychological benefits to identifying harmless abnormalities, for example, to “confirm” harmlessness and because they considered the knowledge derived from testing reassuring. On the other hand, almost all comments from the harmful group focused on psychological harms. The ambivalent group tended to comment that the relative psychological benefits and harms depended on the context or individual preferences, for example, an individual’s predisposition to anxiety, ability to interpret information, or whether they like “knowing” things about their body.

#### C2. Reasons to identify harmless abnormalities

Participants from mostly the valuable group, but also the ambivalent and harmful groups, described reasons for wanting to identify harmless abnormalities. Some described a generalized belief that it is “good to be informed.” Some linked testing information to improved health, for example, commenting that knowing about abnormalities can aid in optimizing one’s body. Some valued identifying abnormalities because this would assist the future management of these, for example, so abnormalities could be discussed and dismissed in health consultations, so abnormalities could be monitored, or because the abnormality could either “become harmful” or advances in medicine would lead to it being reclassified as harmful in the future.

#### C3. Reasons to avoid identifying harmless abnormalities

Participants described reasons to avoid finding harmless test abnormalities, representing comments from half the “harmful” groups and a minority of comments from the other groups. Almost all the comments described the potential for subsequent low-value care as a reason for avoiding identification, for example, overinvestigation and being put through further needless tests, the risks of unnecessary treatment, and other downsides such as costs and wasted time.

#### C4. Context is key for judging value of identifying harmless abnormalities

Participants described ways that their attitudes to finding harmless abnormalities depended on context. Almost half the ambivalent group’s comments were coded to this theme, as well as a minority of comments from the other groups. Some participants indicated that value would depend on the type of abnormality. For example, they wanted to know about abnormalities such as lumps or cardiac irregularities if they were present but accepted leaving less “scary” abnormalities unidentified. Others focused on whether the test was believed to be risky or not, for example, accepting blood tests but not biopsies or radiation from X-rays. Other participants could not understand how finding a harmless abnormality could cause either benefit or harm.

### Interpretative Analysis

Interpretative results examined the implicit beliefs underlying explicit explanations of attitudes to finding harmless abnormalities. They are reported in narrative form to highlight differences between groups. Exemplary comments are in Table 3.

#### I1. Beliefs common across groups

Participants across all groups seemed to believe that knowing as much as possible about one’s own body is important. Furthermore, participants across all groups seemed to believe that tests provide such information reliably, that is, in identifying and distinguishing abnormalities from “normal” findings. Finally, all groups seemed skeptical about the concept of a “harmless” abnormality, although the strength of this skepticism varied between groups.

#### I2. Valuable group was more skeptical about “harmlessness” and attached greater value to information derived from tests

The valuable group emphasized 2 beliefs. The first was a greater skepticism that abnormalities can be “harmless.” Harmlessness appeared to be understood by many in the valuable group as temporary, limited, or suspect. Some participants described that harmless abnormalities could become harmful or wanted to “monitor” abnormalities (temporary harmlessness). Some believed that the abnormality may indicate another harmful pathology or that the true harms of the abnormality may be discovered in the future with the advancement of medical knowledge (limited harmlessness). In response to our explanation that the abnormality would never cause symptoms or be known to the patients, some participants commented that they could not imagine how an abnormality could be “harmless,” and many described valuing tests because they provide “reassurance about symptoms” (suspect harmlessness).

The second belief was that knowledge derived from tests is worthwhile for many reasons—some of which have nothing to do with ruling out pathology—and gaining this knowledge is worth the downsides. While all groups were interested in knowing about their own bodies, the valuable group emphasized this more strongly. They emphasized the value of testing-derived information for shared decision making and reassurance. They also just wanted to “know things,” without necessarily having specific ideas about how they would use that knowledge. Unlike the ambivalent and harmful groups, they deemphasized the downsides associated with gaining this information using testing, such as risks, costs, and inconvenience.

#### I3. Ambivalent and harmful groups were more accepting of “harmlessness,” more skeptical about medical care, and worried about coping

Participants in the ambivalent and harmful groups differed from the valuable group by commonly expressing any of 3 beliefs.

First, it was less common for them to question the harmlessness of abnormalities. When they did question it, they tended to be less strong in their repudiation of harmlessness. Some participants in the ambivalent and harmful groups expressed beliefs that abnormalities can truly be harmless, and therefore it was fine to leave them unidentified.

Second, they expressed distrust in doctors who requested the tests and in the health care system, for example, that some testing was motivated by profiteering and that abnormalities would be inappropriately investigated or treated by some doctors.

Third, they focused on the limitations of their own coping abilities. For example, some expressed concerns that they would hyperfocus on the abnormality, struggle to accept that it is truly harmless, and become anxious as a result of identification.

Therefore, although participants in the ambivalent and harmful groups also expressed concerns about having harmless abnormalities in their body, they believed that the risk these abnormalities posed if left undiscovered was less than the risk of harm from identifying them. The difference between the ambivalent and harmful groups was largely that the ambivalent group described harms as contextual, while the harmful group described them as relatively persistent.

### Quantitative Findings

Table 4 reports the univariate associations between attitudes toward identifying harmless abnormalities (harmful, ambivalent, valuable) and responses to other fixed-response questions.

**Table 4:** Univariate Associations with Attitudes to Identifying Harmless Abnormalities (*N* = 655)^a^

	Harmful, *n* = 129 (19.7%)	Ambivalent, *n* = 160 (24.4%)	Valuable, *n* = 366 (55.9%)	*P* Value*
State/territory of residence				0.65
Australian Capital Territory	6 (33.3)	5 (27.8)	7 (38.9)	
New South Wales	25 (17.0)	36 (24.5)	86 (58.5)	
Northern Territory	3 (30.0)	0	3 (50.0)	
Queensland	25 (22.5)	27 (24.3)	59 (53.2)	
South Australia	5 (13.5)	9 (24.3)	23 (62.2)	
Tasmania	6 (26.1)	6 (26.1)	11 (47.8)	
Victoria	45 (18.6)	63 (26.0)	134 (55.4)	
Western Australia	14 (19.7)	14 (19.7)	43 (60.6)	
Gender				0.33
Female	70 (22.3)	82 (26.1)	162 (51.6)	
Male	55 (17,4)	73 (23.0)	189 (59.6)	
Transgender	0	1 (16.7)	5 (83.3)	
Other gender	2 (15.4)	4 (30.8)	7 (53.9)	
Prefer not to answer	2 (40.0)	0	3 (60.0)	
Age^b^				***<0.001***
	47.8 (18.0)	55.9 (18.8)	56.6 (18.5)	
Have children				0.17
Yes	65 (17.2)	95 (25.2)	217 (57.6)	
No	64 (23.2)	64 (23.2)	148 (53.6)	
Remoteness of residence				0.94
Metropolitan	82 (18.5)	106 (25.2)	232 (55.2)	
Regional	43 (19.6)	51 (23.3)	125 (57.1)	
Remote	4 (25.0)	3 (18.8)	9 (56.3)	
Is or was a health care worker				***0.006***
Yes	40 (30.5)	29 (22.1)	62 (47.3)	
No	88 (17.4)	127 (25.2)	290 (57.4)	
No answer/unclear	1 (5.3)	4 (21.1)	14 (73.7)	
Highest level of education attained				***0.002***
Secondary school incomplete	4 (14.8)	7 (25.9)	16 (59.3)	
Secondary school completed	8 (11.9)	12 (17.9)	47 (70.2)	
Higher learning certificate/diploma	13 (10.8)	29 (24.2)	78 (65.0)	
Undergraduate degree	40 (18.7)	56 (26.2)	118 (55.1)	
Postgraduate degree	64 (28.3)	56 (24.8)	106 (46.9)	
Weekly household income (A$)				***0.002***
Low (<1,000)	26 (14.9)	40 (23.0)	108 (62.1)	
Mid (1,000–<3,000)	40 (21.1)	48 (25.3)	102 (53.7)	
Mid-high (3,000–<4,000)	12 (34.3)	11 (31.4)	12 (34.3)	
High (4,000+)	15 (41.7)	8 (22.2)	13 (36.1)	
Self-rated health				***0.141***
Excellent	12 (24.5)	14 (28.6)	23 (46.9)	
Very good	54 (24.3)	52 (23.4)	116 (52.3)	
Good	45 (19.4)	55 (23.7)	132 (56.9)	
Fair	12 (10.4)	28 (24.4)	75 (65.2)	
Poor	6 (16.2)	11 (29.7)	20 (54.1)	
The more I know about my body the better				***<0.001***
Strongly agree	51 (11.7)	95 (21.8)	289 (66.4)	
Somewhat agree	53 (29.9)	56 (31.6)	68 (38.4)	
Neither	25 (61.0)	9 (22.0)	7 (17.1)	
How commonly are harmless abnormalities unnecessarily treated?				***<0.001***
Commonly	104 (26.9)	78 (20.2)	204 (52.9)	
Uncommonly	16 (8.9)	48 (26.7)	116 (64.4)	
Don’t know	9 (10.2)	34 (38.6)	45 (51.1)	
Extent that health care professionals can distinguish between harmless/harmful abnormalities detected on tests?				***<0.001***
Usually yes	71 (17.8)	115 (23.9)	295 (61.3)	
Usually not	46 (36.2)	33 (26.0)	48 (37.8)	
Don’t know	12 (25.5)	12 (25.5)	23 (48.9)	
Extent that patients who take the “right steps” can avoid unnecessary treatment on abnormalities detected on tests?				***0.088***
Usually yes	94 (19.1)	118 (24.0)	279 (56.8)	
Usually not	31 (24.0)	27 (20.9)	71 (55.0)	
Don't know	4 (11.4)	15 (42.9)	16 (45.7)	

a Row percentages calculated.

b Age is reported as mean (SD).

* Significance set at *P* < 0.15, with significant values indicated by bolded text.

We identified significant associations (all at *P* < 0.001) between the valuable group and stronger agreement that it is better to know more about one’s body compared with the other 2 groups; a lower estimation compared with the harmful group that harmless abnormalities get unnecessarily treated, and; greater belief compared with the other 2 groups that health care professionals can distinguish between harmful and harmless abnormalities on test results. There were also significant associations between the outcome variable and participants’ age, whether they have been a health care worker, educational attainment, income, and self-rated health. Multicollinearity was not detected between any of the variables, and the assumption of proportionality was met.

Due to the large amount of missing income data, model selection was conducted in 2 cohorts: 1) those who answered all questions including income (*n* = 435) and 2) those who answered all questions but regardless of whether they answered the income question (*n* = 655). In both cohorts, models without the self-rated health variable had better fit compared with the ones that included this variable. In cohort 1, the AIC/BIC for the models were 810/907 with income and self-rated health and 806/887 with income but not with self-rated health. In cohort 2, they were 1,183/1,277 and 1,182/1,259, respectively. Even though we cannot compare between cohort 1 and 2, the pattern of findings remained similar. Therefore, the final model was based on cohort 1, as income was considered to be a significant confounder.

Table 5 reports the variables that remained significant predictors of attitudes to identifying harmless abnormalities in regression analysis adjusted for age, education, income, being a health care worker, and self-rated health. This model was selected over alternate combinations of control variables.

**Table 5:** Predictors of Attitudes to Identifying Harmless Abnormalities (*n* = 432)

	Adjusted OR (95% CI)	*P* Value*
The more I know about my body the better		
Strongly agree^a^	1	
Somewhat agree	3.0 (1.9–4.7)	**<0.001**
Disagree or neutral	9.5 (4.2–21.2)	**<0.001**
How commonly are harmless abnormalities unnecessarily treated?		
Uncommon	1	
Common	1.8 (1.1–2.8)	**0.017**
Don’t know	1.2 (0.6–2.5)	0.64
Extent that health care professionals can distinguish between harmless/harmful abnormalities detected on tests?		
Usually yes^a^	1	
Usually not	1.9 (1.2–3.3)	**0.012**
Don’t know	1.2 (0.5–3.2)	0.69
Extent that patients who take the “right steps” can avoid unnecessary treatment on abnormalities detected on tests?		
Usually yes^a^	1	
Usually not	0.6 (0.4–1.1)	0.095
Don’t know	0.8 (0.3–2.3)	0.65

a Indicates reference category.

* Significance set at *P* < 0.05, with significant values indicated by bolded text.

The results indicated that “valuable” attitudes were associated with stronger agreement that the more one knows about their body the better, a lower estimation about the commonality of harmless abnormalities being overtreated, and a stronger belief that clinicians can distinguish between harmless and harmful abnormalities identified on tests.

## Discussion

We examined attitudes to identifying harmless abnormalities on tests and the beliefs related to these attitudes. Most participants considered it valuable to identify harmless abnormalities. They described benefits including psychological reassurance, insights into their bodies, and improved management or monitoring of the abnormality. Their comments appeared to be underpinned by a skepticism that abnormalities can be truly harmless and beliefs that testing information is useful beyond ruling out pathology. They had stronger beliefs that it is good to know as much as possible about one’s body and greater confidence that harmless abnormalities identified on tests will not get overtreated. Conversely, participants who stated that it was not valuable to identify harmless abnormalities believed that doing so would cause them anxiety and may lead to unnecessary medical interventions. Participants with ambivalent attitudes largely emphasized the decisive role of contextual factors.

### Why Individuals Consider It Valuable to Find Harmless Abnormalities

We identified several explanatory factors. First, individuals may not accept that abnormalities are truly harmless. A similar finding was made previously about attitudes to cancer: individuals struggle to conceptualize a “harmless cancer.”^40^ However, cancer tends to be perceived as uniquely serious,^18,41^ to the extent that individuals may avoid cancer testing from fear of diagnosis.^42^ Therefore, struggling to conceptualize “harmless cancers” may not translate to other “harmless abnormalities.” Our findings are therefore important in showing that individuals tend to synonymize “abnormal” with “diseased” in general, in relation to all kinds of abnormalities they mentioned in their text comments. Even participants who were wary of overtreatment and thought it bad to identify harmless abnormalities tended to question the harmlessness of those abnormalities. Furthermore, our findings contribute early evidence that cognitive dissonance informs the understanding of “harmless abnormalities.” This seems likely in instances in which participants, for example, thought it would be reassuring to monitor harmless abnormalities they would otherwise never become aware of or had seemingly improbable motivations for identifying them, such as anticipating scientific progress.

Second, we identified a range of benefits in addition to ruling out pathology that individuals see in identification. These include beliefs that test results aid understanding and self-management of one’s body, beliefs about psychosocial gains to identification, and beliefs about the value of identification for negotiating medical appointments. These findings add to a mature body of work also showing that individuals test for reassurance, to gain bioinformation,^4,43,44^ as tools for negotiating discussions with doctors,^10^ as well as for reasons that were not major findings in our study (e.g., as social signifiers).^7,22^ Our findings nevertheless build on this knowledge, by indicating that even individuals who are concerned about low-value care and do not wish to know about harmless abnormalities perceive broader benefits in identifying them but avoid doing so for other reasons. Previous findings have been about conditions that affect peoples’ lives, such as cancer^44^ and back pain.^16^ Our study suggests that there are some underlying beliefs about bodily abnormalities that influence testing beliefs regardless of the potential for harm (or lack of).

Third, individuals may want testing-derived knowledge without a specific goal in mind. This supports theories that individuals seek health knowledge out of curiosity as well as to aid decision making,^45^ which is observed, for example, among individuals ordering home-based DNA testing kits.^46^

Finally, individuals may believe they can mitigate the risks associated with identifying the abnormalities. Our participants estimated that the overtreatment of harmless abnormalities identified on tests is common, which was unexpected, since previous studies reported consumer awareness about overtreatment resulting from overtesting to be low.^3,47,48^ Our findings supported previous research indicating that the risk of overtreatment is unlikely to dissuade testing.^44^ Like our study, other research found that individuals tend to believe they can avoid overtesting if they are judicious.^3,49,50^ Our study built on these insights by showing that people may nevertheless be motivated to avoid testing, largely due to their own anxiety or because they distrust that health workers will deal with testing findings appropriately.

### Implications for Practice

Our findings combined with previous research carry several implications for practice.

They suggest that communications about testing choices need to address broader motivations for testing. Focusing on risks of tests against risk of disease^11^ misses much of what matters to individuals. Our participants were willing to accept testing risks, inconvenience, and costs to gain information about something with zero risk. This helps explain why many people resist overtesting messages^3^ and deimplementation^6^ that cites risk/benefit. Instead, communication strategies pairing risk/benefit information with addressing the other factors motivating testing choices should be prototyped and tested to determine whether they are more effective.

Our findings underscore a need to also explain testing risks and benefits themselves better. Common messages, such as that tests have ambiguous results or lead to overtreatment,^3,51^ mismatched our participants’ frameworks for thinking about testing. Changing these frameworks using such messages is undermined by biases and assumptions,^20,52^ including resistance to abnormalities being “harmless” as this study found. Harms of tests are therefore easy to minimize and disregard despite compelling evidence to the contrary. Perhaps using psychologically informed communication techniques to overcome these barriers should be examined.^53^ For example, meta-cognitive strategies that promote reappraising beliefs^54^ or interventions to address the role of fear^55^ driving some low-value care^18^ could be tested.

Our findings about the centrality of anticipated psychological impact in judging the value of testing suggest it may be worthwhile to address this in communications. Evidence shows that many people cope poorly with abnormal findings on tests.^49,56^ Yet the psychological impact of testing is seldom discussed in communications about testing choices.^11^ Having these conversations may be valuable for patients, might be an intuitive way to discuss benefits and harms of tests, and could potentially be an entry point to move on to addressing less intuitive aspects of the testing decision.

### Strengths and Limitations

Our sampling had strengths and limitations. The large sample enabled collection of a breadth of perspectives. We gained a good distribution of responses from men and women, across different age groups, and with a distribution between metropolitan/nonmetropolitan participants similar to the Australian population (73% Australians live in major cities).^57^ However, our sample was overeducated (32% of Australians have degrees),^58^ and those with health care training were overrepresented (5% of Australians have health care training).^59^ We adjusted for these factors in our regression but not in the qualitative analysis. Meta recruitment seems to be effective at gaining representative or partially representative samples.^29^ However, it risks introducing skew, for example, by favoring the recruitment of educated participants.^60^ Participants also self-selected into our study and may have been more interested in the topic than average.

Given that more than 30% of income data were missing, we elected not to impute these values, as doing so would likely violate the MAR assumption and risk introducing bias. This choice ensured that our results remain grounded in the observed data, but it also means that findings technically generalize only to participants with complete income information. To assess robustness, we conducted a sensitivity analysis using the full cohort but excluding income as a predictor, and the similarity of results across approaches provides reassurance. In future studies, we plan to use a modified income question to reduce the likelihood of missing responses and improve generalizability. The survey modality introduced strengths alongside limitations. It facilitated a comparative, mixed-methods design, which added depth to our findings. Conversely, the text response data were “shallower” than would be collected using many other qualitative methods. Some participants appeared to misunderstand the primary question, and the extent to which we managed to identify all who did is unknowable. Question wording influences responses,^61^ and the influence of our wording on our data is unclear. Answers may have differed had we, for example, asked what kinds of tests participants would be willing to undergo to identify harmless abnormalities or used a word other than “abnormality.” The response options had limitations: “valuable” is not the opposite of “harmful.” Nevertheless, patterns in responses identified in our analyses suggest that our study captured meaningful differences in attitudes to identifying harmless abnormalities on tests.

As part of the broader survey, participants were shown the “5 questions to ask your doctor” video, which suggests that individuals should ask about risks, costs, and alternatives when facing medical choices.^62^ This may have primed participants, for example, to overrepresent the extent to which they thought about low-value care. Interestingly, despite priming, most people still considered it valuable to find harmless abnormalities on tests.

## Conclusions

Most participants in this study considered it valuable to identify harmless abnormalities on tests, despite knowing that doing so may lead to overtreatment and other harms. Our study suggests that testing decisions transcend epidemiological considerations, and individuals value tests for a range of other reasons. This helps explain why risk/benefit messaging has a limited effect on reducing unnecessary testing. Strategies seeking to persuade individuals to consider the risks or necessity of tests may need to address the broader range of reasons why individuals value testing (or do not) and explain risks better.

## Supplemental Material

sj-docx-1-mdm-10.1177_0272989X251413288 – Supplemental material for Why Most Australians Consider It Valuable to Find Harmless Abnormalities with Diagnostic Tests: A Mixed-Methods Study Supplemental material, sj-docx-1-mdm-10.1177_0272989X251413288 for Why Most Australians Consider It Valuable to Find Harmless Abnormalities with Diagnostic Tests: A Mixed-Methods Study by Tomas Rozbroj, Ming Hui Hoo, Alexandra Gorelik, Denise A. O’Connor and Rachelle Buchbinder in Medical Decision Making
