# Supplementary material for: Why Most Australians Consider It Valuable to Find Harmless Abnormalities with Diagnostic Tests: A Mixed-Methods Study
Source: Med Decis Making. 2026 Jan 30;46(4):432–47. doi: 10.1177/0272989X251413288 (PMC13062466; doi:10.1177/0272989X251413288)
Supplement: sj-docx-1-mdm-10.1177_0272989X251413288 – Supplemental material for Why Most Australians Consider It Valuable to Find Harmless Abnormalities with Diagnostic Tests: A Mixed-Methods Study [file sj-docx-1-mdm-10.1177_0272989X251413288.docx]

# Appendix

## Appendix 1: GRAMMS

| **Item** | **Guide question / description** | **Location** |
| --- | --- | --- |
| 1. | Describe the justification for using a mixed methods approach to the research question. | Methodology (pp.6-7) |
| 2. | Describe the design in terms of the purpose, priority and sequence of methods. | Participants, sample size and recruitment (pp.5-6) |
| 3. | Describe each method in terms of sampling, data collection and analysis. | Sampling & data collection: as above.  Analysis: Analysis (pp.7-9) |
| 4. | Describe where integration has occurred, how it has occurred and who has participated in it. | Methodology (pp.6-7) |
| 5. | Describe any limitation of one method associated with the present of the other method. | Methodology (pp.6-7) & Limitations (pp. 19-21) |
| 6. | Describe any insights gained from mixing or integrating methods. | Methodology (pp.6-7) & Discussion (pp. 17-19) |
| *The GRAMMS Checklist is from O'Cathain A, Murphy E, Nicholl J. The quality of mixed methods studies in health services research. Journal of Health Services Research & Policy. 2008;13(2):92-8. doi: 10.1258/jhsrp.2007.007074.* | | |

## Appendix 2: variables, recodes and use in analysis

Some variables were recoded following data assessment and to aid regression analysis. We recoded Q1 answers into ‘strongly agree’, ‘somewhat agree’ and ‘neutral or disagree’. This reflected that no respondents strongly disagreed and very few somewhat disagreed or were neutral, and descriptive analysis showed differences in patterns of responses on other variables between participants who selected ‘somewhat’ vs ‘strongly’ agree on Q1. For Q2-6, we collapsed the two groups on either end of Likert scales (e.g. very/somewhat common > ‘common’). Patterns of responses between participants in the two groups on either end were similar. Gender and income variables were collapsed to aid interpretation; income was recoded into the Australian Bureau of Statistics income tax brackets ^1^.

| **#** | **Question text** | **Questionnaire response options** | **Recoded to** | **Use in analysis** |
| --- | --- | --- | --- | --- |
| ***Attitudes to identifying harmless abnormalities*** | | | | |
| 1. | In some cases, diagnostic tests find abnormalities in the body that would not go on to cause any harm. For example, they find lumps, elevated readings or physical changes that would not cause symptoms or complications. If they weren't detected, the patient would never find out that they had them.  How valuable or harmful do you think it is to identify abnormalities that would never harm patients? | Very valuable (5)  Somewhat valuable (4)  Neither valuable nor harmful (3)  Somewhat harmful (2)  Very harmful (1) | Valuable (3)  Ambivalent (2)  Harmful (1) | Comparator in qualitative analysis.  Outcome in quantitative analysis. |
| 2. | Can you please explain why you think this about the extent that it would be valuable or harmful? | <free text> | <free text> | Data for qualitative analysis. |
| ***Broader attitudes*** | | | | |
| 3. | 'The more I know about my body the better'. To what extent do you agree with this statement? | Strongly agree (5)  Somewhat agree (4)  Neither agree nor disagree (3)  Somewhat disagree (2)  Strongly disagree (1) | Strongly agree (3)  Somewhat agree (2)  Neutral or disagree (1) | Predictor in quantitative analysis. |
| 4. | One potential consequence of identifying harmless abnormalities is that some of them go on to get treated, which can cause needless suffering, costs and complications.  How common would you guess it is that people get unnecessary treatment on harmless abnormalities detected via diagnostic tests? | Very common (5)  Somewhat common (4)  Somewhat uncommon (3)  Very uncommon (2)  I don't know (1) | Uncommon (3)  Common (2)  Don’t know (1) | Predictor in quantitative analysis. |
| 5. | When abnormalities are detected via diagnostic tests, to what extent would you guess that healthcare professionals could distinguish between abnormalities that would cause patients symptoms and abnormalities that would not? | In all or almost all cases (5)  In most cases (4)  In a minority of cases (3)  Not at all / almost not at all (2)  I don't know (1) | Usually yes (3)  Usually not (2)  Don't know (1) | Predictor in quantitative analysis. |
| 6. | On average, to what extent would you guess that patients can avoid unnecessary treatments for abnormalities found on tests, as long as they take the 'right' steps? E.g. having discussions with doctors, being informed, making careful decisions, etc. | In all or almost all cases (5)  In most cases (4)  In a minority of cases (3)  Not at all or almost not at all (2)  I don't know (1) | Usually yes (3)  Usually not (2)  Don't know (1) | Predictor in quantitative analysis. |
| ***Demographics*** | | | | |
| 7. | State/territory of residence | Australian Capital Territory  New South Wales  Northern Territory  Queensland  South Australia  Tasmania  Victoria  Western Australia | - | All demographics variables were used in sample classification, and controlled for in regression if they met the significance threshold in univariate analysis. |
| 8. | To which gender do you identify? | Female  Male  Transgender female  Transgender male  Non-binary  Gender not listed <text>  Prefer not to say | Female  Male  Transgender  Other gender  No answer |  |
| 9. | How old are you? | 18-100 or older | - |  |
| 10. | Do you have any children? | Yes  No | - |  |
| 11. | Do you live in a capital city, a regional area or a remote area? | A capital city within my state/territory (such as Hobart or Sydney)  A regional area (such as Alice Springs or the Margaret River)  A remote area (such as Coober Pedy or Mount Isa) | - |  |
| 12. | Do you work as a healthcare professional or did you work as one in the past? | Yes, currently a healthcare professional (please tell us what your job is) <text>  Yes, but no longer work as one (please tell us what your job/s were) <text>  No | Yes  No |  |
| 13. | What is the highest level of education you completed? | Did not complete secondary school  Completed secondary school  Higher learning certificate / diploma (i.e. TAFE)  Undergraduate degree  Postgraduate degree | - |  |
| 14. | What is your household income?  The combined gross (before tax) income of everyone living in your house. It can come from any source, i.e. wages, donations, investments, etc.  Weekly household income is displayed without brackets (yearly household income is displayed in brackets) | Prefer not to say  Negative income  Nil income  $1-$149 ($1-$7,799)  $150-$299 ($7,800-$15,599)  $300-$399 ($15,600-$20,799)  $400-$499 ($20,800-$25,999)  $500-$649 ($26,000-$33,799)  $650-$799 ($33,800-$41,599)  $800-$999 ($41,600-$51,999)  $1,000-$1,249 ($52,000-$64,999)  $1,250-$1,499 ($65,000-$77,999)  $1,500-$1,749 ($78,000-$90,999)  $1,750-$1,999 ($91,000-$103,999)  $2,000-$2,499 ($104,000-$129,999)  $2,500-$2,999 ($130,000-$155,999)  $3,000-$3,499 ($156,000-$181,999)  $3,500-$3,999 ($182,000-$207,999)  $4,000-$4,499 ($208,000-$233,999)  $4,500-$4,999 ($234,000-$259,999)  $5,000-$5,999 ($260,000-$311,999)  $6,000-$7,999 ($312,000-$415,999)  $8,000 or more ($416,000 or more) | No answer / prefer not to say  Low (<1000)  Mid (1000 - <3000)  Mid-high (3000-<4000)  High (4000+) |  |
| 15. | In general, would you say your health is...? | Excellent (5)  Very good (4)  Good (3)  Fair (2)  Poor (1) | - |  |

*1. Drury, B. Australian income tax brackets and rates (2024-25 and previous years). 2024 [cited 2024 October 9th]; Available from: https://www.superguide.com.au/author/barbaradruryauthor.*
